# Creatinine/Cystatin C Ratio as a Surrogate Marker for Sarcopenia in Hepatitis-C-Associated Liver Cirrhosis After Achieving a Sustained Virologic Response

**DOI:** 10.3390/cimb48020222

**Published:** 2026-02-18

**Authors:** Aritoshi Koizumi, Tadashi Namisaki, Akihiko Shibamoto, Takashi Inoue, Shohei Asada, Takuya Matsuda, Satoshi Iwai, Yuki Tsuji, Yukihisa Fujinaga, Norihisa Nishimura, Shinya Sato, Koh Kitagawa, Kosuke Kaji, Akira Mitoro, Kiyoshi Asada, Hiroaki Takaya, Ryuichi Noguchi, Hitoshi Yoshiji

**Affiliations:** 1 Department of Gastroenterology, Nara Medical University, 840 Shijo-cho, Kashihara 634-8522, Nara, Japan; yuring0309@naramed-u.ac.jp (A.K.); a-shibamoto@naramed-u.ac.jp (A.S.); asahei@naramed-u.ac.jp (S.A.); takuya@naramed-u.ac.jp (T.M.); satoshi181@naramed-u.ac.jp (S.I.); tsujih@naramed-u.ac.jp (Y.T.); fujinaga@naramed-u.ac.jp (Y.F.); nishimuran@naramed-u.ac.jp (N.N.); shinyasato@naramed-u.ac.jp (S.S.); kajik@naramed-u.ac.jp (K.K.); mitoroak@naramed-u.ac.jp (A.M.); htky@naramed-u.ac.jp (H.T.); rnoguchi@naramed-u.ac.jp (R.N.); yoshijih@naramed-u.ac.jp (H.Y.); 2 Department of Evidence-Based Medicine, Nara Medical University, 840 Shijo-cho, Kashihara 634-8522, Nara, Japan; tkinoue0@naramed-u.ac.jp; 3 Clinical Research Center, Nara Medical University, 840 Shijo-cho, Kashihara 634-8522, Nara, Japan; kasada@naramed-u.ac.jp

**Keywords:** sarcopenia, liver cirrhosis, creatinine, cystatin C, Cr/CysC ratio, surrogate marker, sustained virological response

## Abstract

The creatinine/cystatin C ratio (CCR) has emerged as a simple surrogate marker for muscle mass. This study aimed to evaluate the clinical utility of CCR in identifying sarcopenia among patients with hepatitis-C-virus-related liver cirrhosis who achieved a sustained virological response following antiviral treatment. In this retrospective study, 111 patients treated at our hospital between 2017 and 2022 were assessed for sarcopenia using the Japan Society of Hepatology criteria, which includes handgrip strength (HGS) and skeletal muscle mass index (SMI) measured via computed tomography. Sarcopenia was diagnosed in 30 patients (27.9%). The median CCR was 0.78 in males and 0.55 in females. Multivariate logistic regression analysis identified CCR < 0.56 as an independent factor associated with sarcopenia. Receiver operating characteristic curve analysis demonstrated good diagnostic performance, with an area under the curve of 0.761 for males and 0.801 for females. Furthermore, overall survival was significantly higher in patients with higher CCR values (>0.65 in males and >0.54 in females). The discriminative ability of CCR was comparable to that of HGS, SMI, and the composite diagnosis of sarcopenia. These findings suggest that CCR is a practical and reliable marker for sarcopenia in this patient population.

## 1. Introduction

Hepatitis C virus (HCV) infection is a major cause of liver cirrhosis globally, accounting for approximately 20–30% of cases worldwide [1] and contributing substantially to cirrhosis-related morbidity and mortality, despite major advances in antiviral therapy [2,3]. The advent of direct-acting antivirals has enabled sustained virological response (SVR) rates exceeding 95%, significantly reducing liver-related morbidity and mortality. However, although viral eradication improves hepatic outcomes, LC-related complications such as malnutrition and sarcopenia continue to pose significant clinical challenges [4].

Sarcopenia is defined as a progressive, generalized loss of skeletal muscle mass and strength; it is seen in 30–70% of patients with LC and is recognized as an independent risk factor for poor prognosis [5,6]. Sarcopenia increases the risk of hepatic decompensation, infections, hepatic encephalopathy, and mortality. In the Japan Society of Hepatology (JSH) criteria [7,8,9], sarcopenia is recognized not only as an age-related condition, but it can also be secondary to chronic diseases such as LC and hepatocellular carcinoma [3].

The accurate diagnosis of sarcopenia requires assessments of both muscle mass and muscle strength [6]. Techniques such as computed tomography (CT) and dual-energy X-ray absorptiometry (DEXA) are used to evaluate muscle mass [5], while handgrip dynamometry is commonly used to assess muscle strength. However, these measurements are not always feasible in daily practice due to the limited availability of equipment, patient burden, cost, and logistical constraints [5].

There is a growing demand for simple and accessible surrogate markers that can reliably reflect skeletal muscle mass. Creatinine (Cr) is a metabolite generated by muscle turnover and correlates with muscle mass [10], while cystatin C (CysC) is unaffected by muscle mass but reflects glomerular filtration. Accordingly, the Cr/CysC ratio (CCR) can be used to estimate muscle mass relative to renal function. CCR theoretically adjusts for muscle mass estimation by controlling for renal clearance. A low CCR suggests reduced muscle mass, even if kidney function is preserved. Previous studies have demonstrated that the CCR is associated with sarcopenia—characterized by both reduced muscle mass and impaired muscle function—in community-dwelling older adults [11], patients with diabetes [12], individuals with chronic kidney disease [13], and those with malignancies [14]. but its clinical relevance remains unclear in patients with LC, particularly in those that have achieved SVR.

This study aimed to investigate whether the CCR is associated with sarcopenia in patients with HCV-related LC who achieved SVR, as well as to determine its diagnostic performance and optimal cutoff value for clinical use.

## 2. Patients and Methods

### 2.1. Study Design and Population

**Figure 1:**
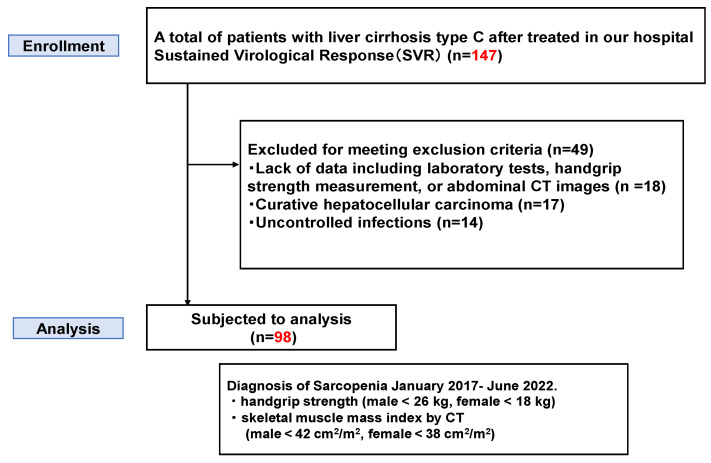
Flowchart of patient enrollment and analysis.

This single-center retrospective study initially enrolled 160 consecutive patients with HCV-related liver cirrhosis who achieved SVR after direct-acting antiviral therapy between January 2017 and June 2022 at Nara Medical University. Liver cirrhosis was diagnosed based on a combination of clinical signs, laboratory findings (e.g., thrombocytopenia, hypoalbuminemia), and radiological features, including liver surface nodularity, splenomegaly, and collateral circulation on imaging. Patients were excluded if they had missing key data (e.g., laboratory tests, handgrip strength [HGS] measurements, or abdominal CT scans) (*n* = 18), curative hepatocellular carcinoma (*n* = 17), and uncontrolled infections (*n* = 14), After applying the exclusion criteria, 111 patients were eligible for analysis (Figure 1). CT, HGS, and laboratory measurements were performed at a stable post-SVR time point, primarily during routine follow-up or Hepatocellular carcinoma surveillance. All evaluations were conducted after achievement of SVR, with a median interval of 3 months (SVR12: sustained virologic response at 12 weeks) (IQR 1–4) between SVR and evaluation.

Among 147 patients with HCV-related liver cirrhosis who achieved SVR, 98 were included in the final analysis after excluding those with missing data, hepatocellular carcinoma, or uncontrolled infections. Sarcopenia was diagnosed based on handgrip strength and skeletal muscle index (SMI) measured via CT between January 2017 and June 2022.

### 2.2. Diagnosis of Sarcopenia

Sarcopenia was diagnosed based on the presence of low skeletal muscle mass and low muscle strength, according to the JSH criteria [6]. HGS was measured in the dominant hand using a calibrated dynamometer [3]. Each participant performed two measurements, and the maximum value was used for analysis. CT-based SMI was assessed at the level of the third lumbar vertebra (L3). CT image analysis was conducted by independent reviewers blinded to clinical information. Briefly, skeletal muscle area was quantified on a single axial CT slice at L3, including the psoas, paraspinal, and abdominal wall muscles. Muscle segmentation was performed using dedicated image analysis software with predefined Hounsfield unit thresholds for skeletal muscle. To minimize variability, CT images with comparable slice thickness were selected for analysis. Measurements were independently performed by two blinded reviewers, and both inter- and intra-rater reliability were evaluated using intraclass correlation coefficients (ICC), which demonstrated excellent agreement.

### 2.3. Laboratory Measurements

Serum Cr and CysC were measured on the same day as the CT scan and HGS. The CCR was calculated by dividing the serum Cr (mg/dL) by the serum CysC level (mg/L) [15]. Serum creatinine was measured using an enzymatic method, and cystatin C was assessed by immunoassay with automated analyzers.

Other laboratory parameters were determined according to standard procedures at our institutional laboratory, employing automated analyzers and commercially available assays.

The Child–Pugh score was calculated based on serum bilirubin, serum albumin, prothrombin time, ascites, and hepatic encephalopathy.

The albumin–bilirubin (ALBI) score was calculated using the following formula:

ALBI score = (log10 bilirubin [μmol/L] × 0.66) + (albumin [g/L] × −0.085),

Patients were subsequently classified according to ALBI and modified ALBI grades.

The fibrosis-4 (FIB-4) index was calculated as follows:

FIB-4 = (age × AST)/(platelet count × √ALT).

Branch-chain amino acid to Tyrosine Ratio (BTR)was determined as (valine + leucine + isoleucine)/tyrosine, measured using an amino acid analyzer. It reflects hepatic functional reserve and protein–energy malnutrition in cirrhosis.

### 2.4. Statistical Analysis

Continuous variables are expressed as the mean ± standard deviation or median (interquartile range [IQR]) and compared using the student’s *t*-test or Mann–Whitney U test. Categorical variables are expressed as frequencies and percentages and compared using the chi-square or Fisher’s exact test. Univariate and multivariate logistic regression analyses were performed to identify predictors of sarcopenia. Receiver operating characteristic (ROC) curves were constructed to evaluate the diagnostic performance of CCR. Sex-specific area under the curve (AUC) values were calculated. The optimal cutoff values were determined using the Youden index. Statistical significance was set at *p* < 0.05. Statistical analyses were performed using IBM SPSS Statistics 29.0 (https://www.ibm.com/products/spss (accessed on 1 January 2020)). Given known physiological sex differences in muscle mass and creatinine generation, sex-specific CCR cutoffs were prespecified as the primary analytic approach. Thresholds were determined using ROC analyses stratified by sex. Because the number of explanatory variables (four) was relatively large in relation to the number of events in the logistic regression model, a penalized regression approach was adopted to mitigate overfitting. Specifically, multivariable analysis was performed using ridge regression, with sex included as a covariate. In addition to dichotomized analyses, CCR was also modeled as a continuous variable. Statistical significance was defined as a two-sided *p* value < 0.05.

## 3. Results

**Figure 2:**
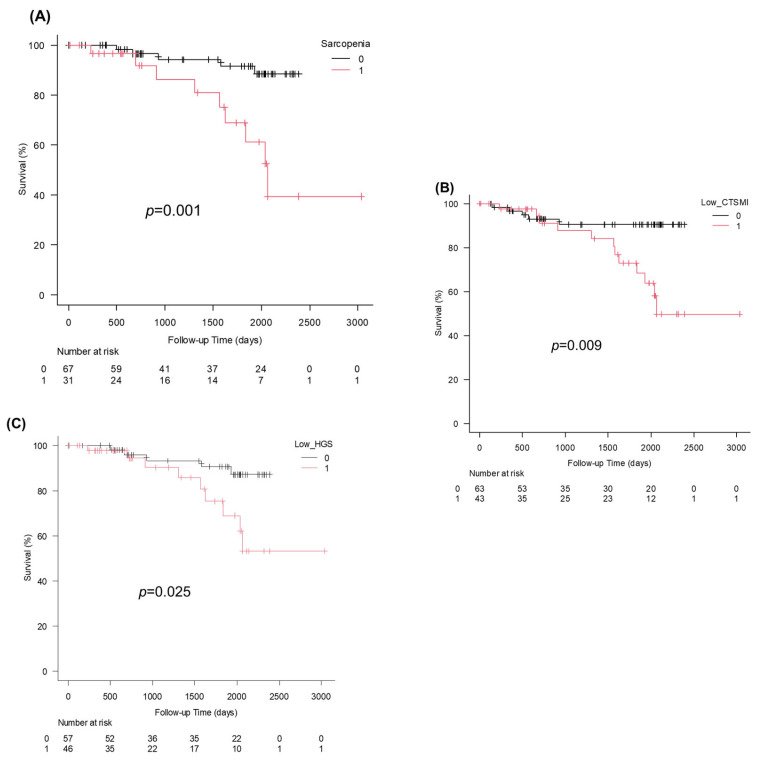
(**A**) Kaplan–Meier survival curves stratified by sarcopenia status. Patients without sarcopenia demonstrated significantly better survival compared with those with sarcopenia (*p* = 0.001). (**B**) Kaplan–Meier survival curves stratified by computed tomography (CT)-defined skeletal muscle index (SMI). Patients with low SMI had a significantly worse prognosis than those with normal SMI (*p* = 0.009). (**C**) Kaplan–Meier survival curves stratified by handgrip strength (HGS). Patients with low HGS had significantly poorer survival compared with those with normal HGS (*p* = 0.025).

The analysis included 98 patients (45.9% male) with a mean age of 71.2 ± 6.5 years. The mean Child–Pugh score was 6.0 ± 1.5, with 93 (94.9%), 3 (3.1%), and 1 (1.0%) patients being classified as Child–Pugh grades A, B, and C, respectively (Table 1). Regarding the modified albumin–bilirubin (m-ALBI) grade, 66 (67.3%), 15 (15.3%), 13 (13.2%), and 4 (4.1%) patients were classified as grade 1, 2a, 2b, and 3, respectively. Moreover, the mean fibrosis-4 (FIB-4) index was 5.0 ± 3.3. The median CCR was 0.76 ± 0.25 in males and 0.57 ± 0.12 in females. Sarcopenia was diagnosed in 31 patients (31.6%), more frequently in females. Males with sarcopenia had significantly lower hemoglobin, CCRs, and HGS versus those without sarcopenia. Meanwhile, females with sarcopenia had significantly lower CCRs and SMI versus those without sarcopenia. Prognosis was significantly better in patients without sarcopenia, those with normal CT-SMI findings, and those with normal HGS (Figure 2).

### 3.1. CCR and Sarcopenia

**Figure 3:**
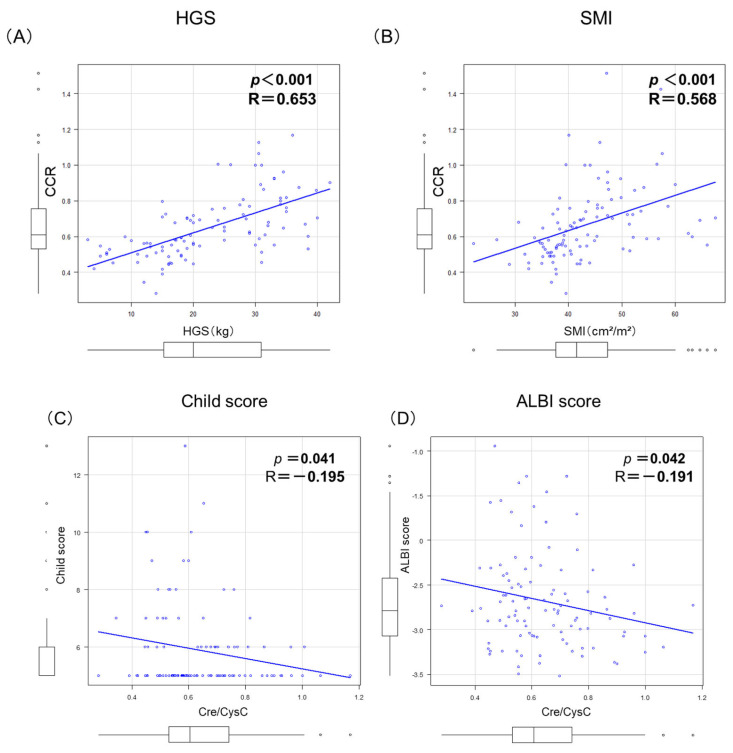
Correlations between creatinine/cystatin C ratio (CCR) and indicators of sarcopenia and between CCR and liver function scores. Significant positive correlations were observed btween (**A**) CCR and handgrip strength (HGS) and between (**B**) CCR and skeletal muscle index (SMI). Significant inverse correlations were observed between (**C**) CCR and Child–Pugh score and between (**D**) CCR and albumin–bilirubin (ALBI) score.

The CCR significantly correlated with both HGS (R = 0.664, *p* < 0.001) and SMI (R = 0.579, *p* < 0.001) (Figure 3A,B), while it demonstrated a fair negative correlation with Child–Pugh score (R = −0.193, *p* = 0.045) and m-ALBI score (R = −0.201, *p* = 0.047) (Figure 3). On the ROC curve analysis, the CCR had good diagnostic performance at cutoffs of 0.65 in males (AUC: 0.751, 95% CI: 0.542–0.890) and 0.54 in females (AUC: 0.799, 95% CI: 0.645–0.932) (Figure 3C,D). Sex-stratified ROC analyses identified optimal CCR cutoffs of 0.65 in males and 0.54 in females. These thresholds were subsequently applied in the primary survival analyses.

### 3.2. Risk Factor Analysis

**Figure 4:**
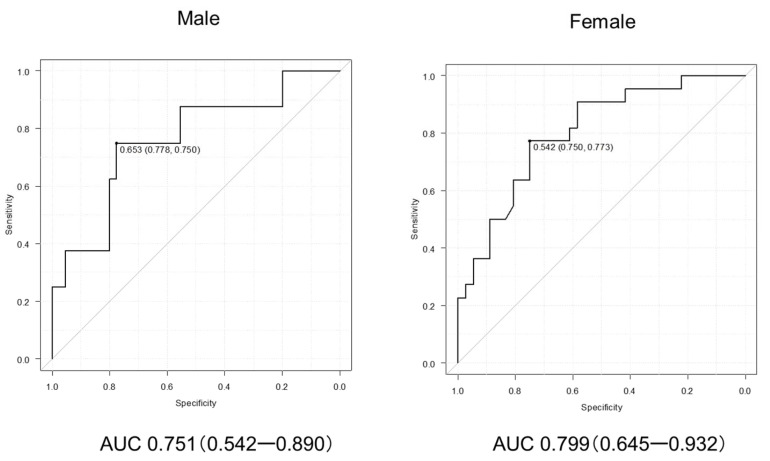
Receiver operating characteristic (ROC) curves of creatinine/cystatin C ratio (CCR) for predicting sarcopenia in male and female patients with liver cirrhosis who achieved a sustained virological response (SVR) following treatment for hepatitis C virus (HCV) infection. The area under the curve (AUC) was 0.751 (95% CI: 0.542–0.890) in males and 0.799 (95% CI: 0.645–0.932) in females.

Based on the ROC curve analysis, the optimal CCR cutoff value was 0.65 in males and 0.54 in females (Figure 4). On univariate logistic regression analysis, the optimal CCR cutoff value was 0.65 in males and 0.54 in females was significantly associated with sarcopenia (OR: 6.69, 95% CI: 2.62–17.10, *p* < 0.001), and this remained an independent predictor of sarcopenia in the multivariate logistic regression model (OR: 5.26, *p* < 0.001) (Table 2). The proportional hazards assumption was evaluated using Schoenfeld residuals in the multivariable Cox model adjusted for sex and hemoglobin (Table 3). All covariates had *p* values ≥ 0.05, and no significant violations were detected, confirming that the proportional hazards assumption was satisfied.

### 3.3. Prognosis of Patients with Cirrhosis According to CCR

**Figure 5:**
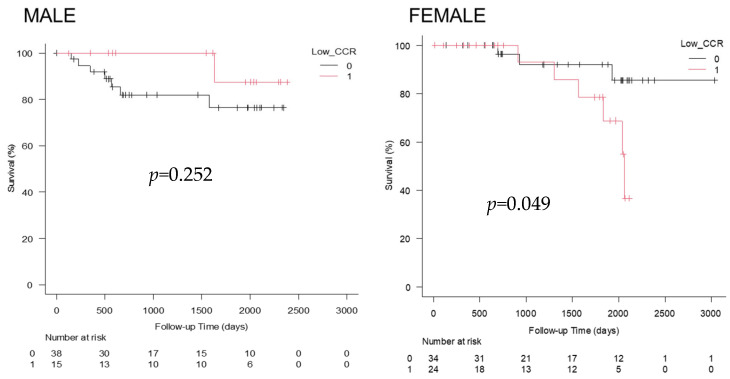
Kaplan–Meier survival curves stratified by serum creatinine/cystatin C ratio (CCR). No significant differences in survival were seen among male patients with CCR ≥ 0.65 compared with CCR < 0.65 (*p* = 0.252). In contrast, female patients with CCR ≥ 0.54 demonstrated significantly better survival compared with those with CCR < 0.54 (*p* = 0.049). These analyses were conducted in patients with liver cirrhosis who achieved sustained virological response (SVR) following treatment for hepatitis C virus (HCV) infection.

Kaplan–Meier survival analyses were performed using sex-specific CCR cutoffs (>0.65 vs. ≤0.65 in males; >0.54 vs. ≤0.54 in females). On Kaplan–Meier analysis, there was significant difference in prognosis on follow-up when patients were stratified by CCR. In particular, survival was significantly better in females with CCR ≥ 0.54 versus CCR <0.54 (Figure 5). However, no significant difference in survival was observed between male patients with CCR ≥ 0.65 and those with CCR < 0.65.

### 3.4. Association Between Skeletal Muscle Index (SMI), Handgrip Strength (HGS), and Sarcopenia

In patients with HCV-related liver cirrhosis who achieved SVR, sarcopenia, low HGS, and low SMI all demonstrated comparable diagnostic performance in predicting adverse clinical outcomes (Figure S1 and Table S1).

## 4. Discussion

This study demonstrated that CCR is strongly associated with sarcopenia in patients with HCV-related LC who achieved SVR, representing one of the first studies to validate CCR as a practical surrogate marker for sarcopenia in this population [16]. The rationale for using the CCR lies in its biological basis. Serum Cr is derived from muscle metabolism and correlates with muscle mass, whereas serum CysC is unaffected by muscle mass and reflects glomerular filtration. Therefore, a low CCR indicates reduced muscle mass relative to renal function. The CCR has been used as a surrogate marker for sarcopenia, rather than serving solely as an indicator of muscle mass [17].

Additionally, the AUC values of 0.761 in males and 0.801 in females suggest acceptable diagnostic performance, and the CCR cutoff of <0.56 aligns with thresholds proposed in other clinical contexts.

Sarcopenia has emerged as a critical extrahepatic manifestation of chronic liver disease, reflecting not only nutritional decline, but also systemic inflammation, endocrine dysregulation, and impaired hepatic synthetic function [18]. Notably, in the post-SVR setting where hepatic inflammation is resolved and fibrosis progression is arrested, sarcopenia may persist or even worsen, potentially due to irreversible metabolic derangements or ongoing mitochondrial dysfunction [19]. Therefore, the continued assessment of muscle mass and strength remains clinically relevant even after viral eradication. The prognostic significance of sarcopenia is well-established across multiple etiologies of cirrhosis, and our findings further support its relevance even after HCV eradication [20]. In our cohort, patients with sarcopenia had significantly worse survival rates, which aligns with prior studies that identified muscle wasting as an independent predictor of mortality regardless of virologic response [21]. Clinically, these results emphasize the importance of continued nutritional and physical assessment in patients with HCV-related cirrhosis, even after SVR is achieved. Incorporating sarcopenia screening into routine follow-up protocols can help facilitate early interventions such as dietary optimization, resistance training, and hepatology-directed rehabilitation programs [22]. Additionally, patients with low HGS or SMI may need closer surveillance for hepatic events or even be considered for transplantation in selected cases.

HGS is a simple, cost-effective, and reproducible measure of muscle function that has been linked to liver-related mortality and hospitalization risk. Conversely, the SMI is widely considered as an objective standard for accurately quantifying muscle mass [23], but its reliance on imaging, typically via CT, limits its routine use in certain clinical settings. In our study, both HGS and SMI exhibited diagnostic performance comparable to the established sarcopenia criteria, indicating their effectiveness as standalone markers in appropriate clinical contexts [24]. Interestingly, the similar AUC values observed for sarcopenia, low HGS, and low SMI suggest that combination of the two may not significantly enhance diagnostic accuracy compared to using each marker individually [25]. Nevertheless, the clinical context should guide which marker to prioritize. For instance, in patients with LC, HGS may be disproportionately reduced compared to SMI, thereby potentially underestimating muscle mass loss [26]. Conversely, patients with preserved muscle mass but reduced muscle quantity may still have an increased risk for complications due to impaired metabolic reserve [27]. Additionally, patients with low HGS or SMI may need closer surveillance for hepatic events or even consideration for transplantation in selected cases [28]. Importantly, although the composite diagnosis of sarcopenia includes both low HGS and low SMI, neither individual component was inferior in prognostic utility, suggesting that either marker can be a feasible alternative in resource-limited settings [29]. Thus, all three indicators (i.e., sarcopenia, low HGS, and low SMI) demonstrated comparable diagnostic performance in predicting adverse clinical outcomes, including hepatic decompensation and mortality [24].

There were notable sex-specific differences in our analysis, particularly regarding the optimal CCR cutoff values. Consistent with previous studies, males had a higher CCR threshold (0.65) compared to females (0.54), likely reflecting the inherent sex-based physiological differences in muscle mass distribution [26]. Thus, sex-specific criteria should be considered when evaluating sarcopenia, because using uniform cutoff values can underestimate or overestimate muscle loss in certain populations. Recognizing these differences is essential for accurate risk stratification and for guiding appropriate clinical interventions [30].

Despite the strengths of this study, several limitations must be considered. First, the retrospective design and single-center setting may restrict the generalizability of our findings. Second, the sample size was very small, thus limiting the statistical power for subgroup analyses. Third, the observational design precludes an analysis of causality. Fourth, other confounding factors such as dietary intake and physical activity were not assessed. Finally, sarcopenia was assessed at a single time point, and thus, longitudinal changes in muscle status and their dynamic relationship with outcomes were not evaluated. Given the practical challenges of CT and DEXA scans in routine practice, the CCR offers a convenient and cost-effective alternative for initial screening. Thus, patients with low CCR can be prioritized for further muscle mass assessment and early intervention, including nutritional support and exercise programs.

## 5. Conclusions

In conclusion, the CCR is a simple and practical surrogate marker for sarcopenia in patients with HCV-associated LC who achieved SVR. CCR < 0.56 was an independent predictor of sarcopenia with good diagnostic accuracy. However, multicenter prospective validation studies are needed to establish standardized cutoff values across different ethnicities and etiologies of cirrhosis. Nevertheless, the routine evaluation of CCR can enable gastroenterologists and primary care providers to stratify the risk of sarcopenia with minimal additional cost.

## Figures and Tables

**Table 1:** Clinical characteristics of patients with cirrhosis with and without sarcopenia.

Variables	All Patients (*n* = 98)	Sarcopenia (Group S) (*n* = 31)	Non-Sarcopenia (Group NS) (*n* = 67)	*p* Value ^a^
Age, Years	71.2 ± 6.5	72.3 ± 10.3	69.0 ± 9.0	0.78
Sex, female/male	53/45	24/7	29/38	0.002
Child–Pugh grade A/B/C	93/3/1	28/2/0	64/1/1	0.485
Child–Pugh score	6.0 ± 1.5	5.9 ± 1.8	6.0 ± 2.0	0.45
ALBI grade 1/2a/2b/3	63/15/13/4	18/5/6/1	45/10/7/3	0.661
ALBI score	−2.6 ± 0.5	−2.7 ± 0.7	−2.6 ± 0.4	0.51
Ascites	11 (9.9)	7 (22.6)	4 (6.0)	0.033
Gastroesophageal varices	42 (37.8)	14 (45.1)	28 (41.8)	0.32
HGS, kg				
Male	29.3 ± 5.1	21.6 ± 4.7	31.7 ± 5.3	<0.001
female	14.3 ± 4.6	14.7 ± 1.9	14.0 ± 3.0	0.77
SMI, cm^2^/m^2^				
male	43.0 ± 5.1	41.3 ± 2.8	49.1 ± 5.7	0.002
female	39.0 ± 5.5	37.3 ± 2.1	41.0 ± 5.1	<0.001
BCAA supplementation	30 (27.0)	6 (19.4)	24 (35.8)	0.76
L-carnitine supplementation	3 (2.7)	0 (0)	3 (4.5)	0.30
Diuretics	26 (23.4)	10 (32.3)	16 (23.9)	0.55
Cr/cystatin ratio	0.69 ± 0.20	0.53 ± 0.14	0.73 ± 0.18	<0.001
male	0.76 ± 0.25	0.62 ± 0.19	0.80 ± 0.31	0.017
female	0.57 ± 0.12	0.51 ± 0.10	0.60 ± 0.13	<0.001
Hemoglobin, g/dL				
male	12.9 ± 2.2	11.5 ± 0.8	13.4 ± 2.2	0.03
female	12.2 ± 1.8	11.6 ± 2.3	12.2 ± 1.9	0.099
MCV, fL	93.8 ± 7.4	92.1 ± 6.8	95.3 ± 9.3	0.34
MCH, pg	30.9 ± 0.9	30.7 ± 3.2	31.1 ± 3.6	0.77
MCHC, %	32.6 ± 0.7	32.9 ± 0.9	33.0 ± 0.8	0.65
Platelet, ×10^4^/μL	12.4 ± 5.1	12.2 ± 3.5	12.1 ± 4.9	0.67
Albumin, g/dL	4.0 ± 0.6	4.0 ± 0.5	4.1 ± 0.2	0.44
PT, %	86.6 ± 8.1	83.4 ± 16.7	88.0 ± 15.3	0.49
Total bilirubin, mg/dL	1.0 (0.7–1.4)	1.0 (0.7–1.3)	1.1 (0.7–1.4)	0.88
ChE, U/L	218.6 ± 77.1	205.4 ± 37.7	224.5 ± 87.0	0.28
BTR	4.9 ± 1.9	5.0 ± 2.2	4.8 ± 1.9	0.61
NH3, μg/dL	50.9 ± 31.0	61.3 ± 41.3	40.8 ± 26.9	0.75
BUN, mg/dL	17.8 ± 9.0	18.0 ± 12.2	17.5 ± 7.7	0.32
Creatinine, mg/dL	0.7 (0.6–1.0)	0.7 (0.6–0.9)	0.8 (0.7–1.0)	0.56
Zinc, μg/dL	70.5 ± 14.8	67.3 ± 11.5	73.8 ± 15.0	0.32
FIB4 index	5.0 ± 3.4	4.9 ± 3.2	5.0 ± 3.4	0.84
P-III-P, U/mL	0.9 ± 0.5	0.9 ± 0.5	0.9 ± 0.5	0.32
7S domain of type IV collage, ng/mL	7.2 (4.2–10.2)	7.0 (5.3–9.6)	7.4 (4.5–9.0)	0.62
CRP, mg/dL	0.1 (0.02–0.32)	0.1 (0.02–0.32)	0.1 (0.01–0.20)	0.23

*p* values state Sarcopenia vs. non-Sarcopenia. Data are presented as mean ± SD or median (IQR). “^a^” denotes the statistical test used (now explicitly defined).

**Table 2:** Risk factors for sarcopenia in patients with liver cirrhosis.

Variables (*n* = 31)	Number of Patients *n* (%)	Univariate Analysis		Multivariate Analysis *	
		OR (95% CI)	*p*-Value	OR	*p*-Value
Age ≥ 70 years	23 (74.2)	1.71 (0.67–4.40)	0.265		
Sex: Male	7 (22.6)	0.22 (0.08–0.59)	0.002	0.27	0.003
Child–Pugh grade B or C	2 (6.5)	2.21 (0.30–16.40)	0.440		
mALBI grade 2a	5 (16.7)	1.25 (0.38–3.17)	0.717		
mALBI grade 2b	6 (20.0)	2.14 (0.63–7.26)	0.221		
mALBI grade 3	1 (3.3)	0.83 (0.08–8.55)	0.878		
Presence of ascites	7 (22.6)	3.58 (1.23–17.10)	0.023		
Presence of varices	13 (41.9)	1.44 (0.60–3.48)	0.412		
Hemoglobin <10.8 g/dL for females, <12.4 g/dL for males	15 (48.4)	5.34 (2.02–14.10)	0.001	3.88	0.009
Platelet count < 12.0 × 10^4^/μL	16 (51.6)	1.04 (0.44–2.43)	0.936		
Albumin < 4.0 g/dL	14 (45.2)	2.24 (0.92–5.46)	0.075		
Prothrombin time < 70%	10 (32.3)	1.80 (0.69–4.69)	0.227		
Total bilirubin ≥ 1.5 mg/dL	7 (22.6)	1.10 (0.40–3.09)	0.850		
Cholinesterase < 210 U/L	18 (60.0)	2.17 (0.90–5.22)	0.085		
BTR < 4.6	11 (36.7)	0.72 (0.30–1.75)	0.467		
Ammonia ≥ 40 μg/dL	8 (29.6)	0.60 (0.23–1.57)	0.299		
BUN ≥ 13 mg/dL	22 (71.0)	0.65 (0.24–1.71)	0.379		
Creatinine ≥ 0.6 mg/dL	19 (61.3)	0.19 (0.06–0.54)	0.002		
Zinc < 70 μg/dL	17 (60.7)	5.24 (1.29–8.12)	0.012	2.07	0.098
FIB4 index ≥ 5.0	16 (51.6)	0.60 (0.25–1.41)	0.239		
Type IV collagen 7S domain ≥ 7.0 ng/mL	11 (45.8)	1.69 (0.65–4.41)	0.282		
Ferritin ≥ 150 ng/mL	6 (20.7)	1.59 (0.51–4.99)	0.423		
CRP ≥ 0.5 mg/dL	9 (29.0)	2.91 (1.00–8.51)	0.051		
Cr/cystatin C ratio <0.54 for females, <0.65 for males	21 (67.7)	6.69 (2.62–17.10)	<0.001	5.26	<0.001
Cr/cystatin C ratio (continuous)	—	8.41 × 10^−6^ (3.16 × 10^−8^–2.24 × 10^−3^)	<0.001		

* Ridge logistic regression was employed.

**Table 3:** Follow-up duration, number of deaths/events, and definitions of study endpoints.

	Background Data for Time-to-Death Analysis			Multivariable Cox Regression Analysis	
Grouping factor (total *n* = 98)	Index for higher-risk group	Observation period (days) median and [IQR]	Number of patient deaths	Covariates for multivariable anaysis	Cox regression HR, [95% CI] and *p*-value
CT-SMI [cm^2^/m^2^]	<42 for male and <38 for female	1455 [586, 2038]	*n* = 12 for higher-risk group	Sex and Hb category	HR = 2.54 [0.79, 8.18] and *p* = 0.117
			*n* = 5 for control group		
HGS [kg]	<26 for male and <18 for female		*n* = 9 for higher-risk group	Sex and Hb category	HR = 1.96 [0.55, 6.94] and *p* = 0.298
			*n* = 5 for control group		
Sarcopenia	Having high risks in both SMI and HGS		*n* = 9 for higher-risk group	Sex and Hb category	HR = 3.14 [0.82, 12.09] and *p* = 0.096
			*n* = 5 for control group		
CCR	<0.65 for male and <0.54 for female		*n* = 10 for higher-risk group	Sex and Hb category	HR = 0.94 [0.34, 2.55] and *p* = 0.899
			*n* = 7 for control group		

## Data Availability

The original contributions presented in this study are included in the article and Supplementary Materials. Further inquiries can be directed to the corresponding author.

## Supplementary Materials

The following supporting information can be downloaded at: https://www.mdpi.com/article/10.3390/cimb48020222/s1.

## Abbreviations

The following abbreviations are used in this manuscript:

AC	alcoholic cirrhosis
ALBI	albumin–bilirubin
ALD	alcohol-related liver disease
BIA	bioelectrical impedance analysis
BMI	body mass index
BTR	branched amino acids-to-tyrosine ratio
BUN	blood urea nitrogen
CP	Child–Pugh score
CT	computed tomography
EAA	endotoxin activity assay
FIB-4	fibrosis-4 index
GPI	glycosylphosphatidyl inositol
Hb	hemoglobin
HBV	hepatitis B virus
HCV	hepatitis C virus
HGS	handgrip strength
JSH	Japan Society of Hepatology
LC	liver cirrhosis
MASH	Metabolic-dysfunction-associated steatohepatitis
P-III-P	procollagen N-terminal peptide
SMI	skeletal muscle mass index
