# Supplementary material for: Creatinine/Cystatin C Ratio as a Surrogate Marker for Sarcopenia in Hepatitis-C-Associated Liver Cirrhosis After Achieving a Sustained Virologic Response
Source: Curr Issues Mol Biol. 2026 Feb 18;48(2):222. doi: 10.3390/cimb48020222 (PMC12939024; doi:10.3390/cimb48020222)
Supplement: Supplementary file 1 [file cimb-48-00222-s001.zip › cimb-4110426-supplementary.pptx]

## Slide 1
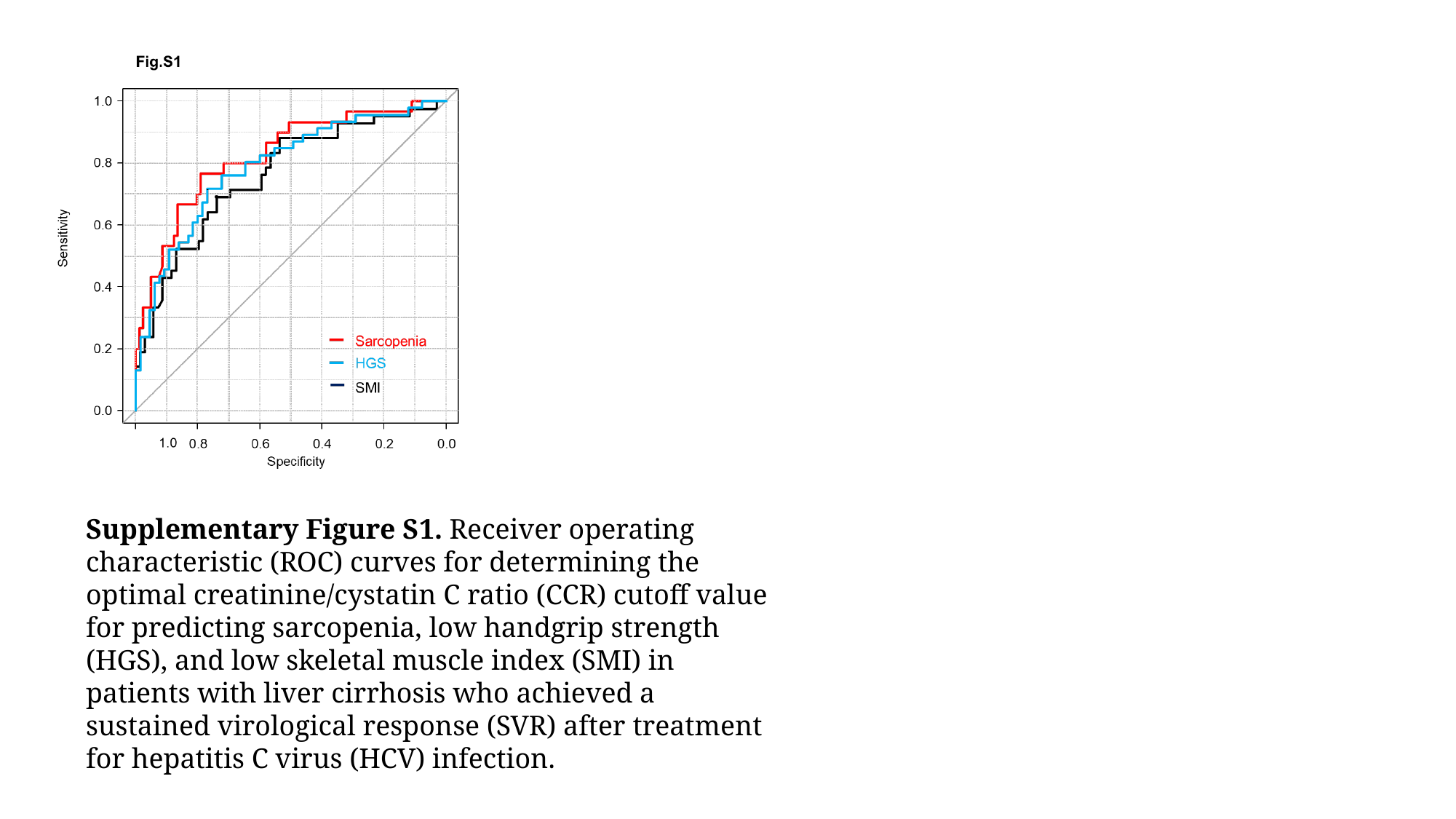

Supplementary Figure S1. Receiver operating characteristic (ROC) curves for determining the optimal creatinine/cystatin C ratio (CCR) cutoff value for predicting sarcopenia, low handgrip strength (HGS), and low skeletal muscle index (SMI) in patients with liver cirrhosis who achieved a sustained virological response (SVR) after treatment for hepatitis C virus (HCV) infection.

## Slide 2
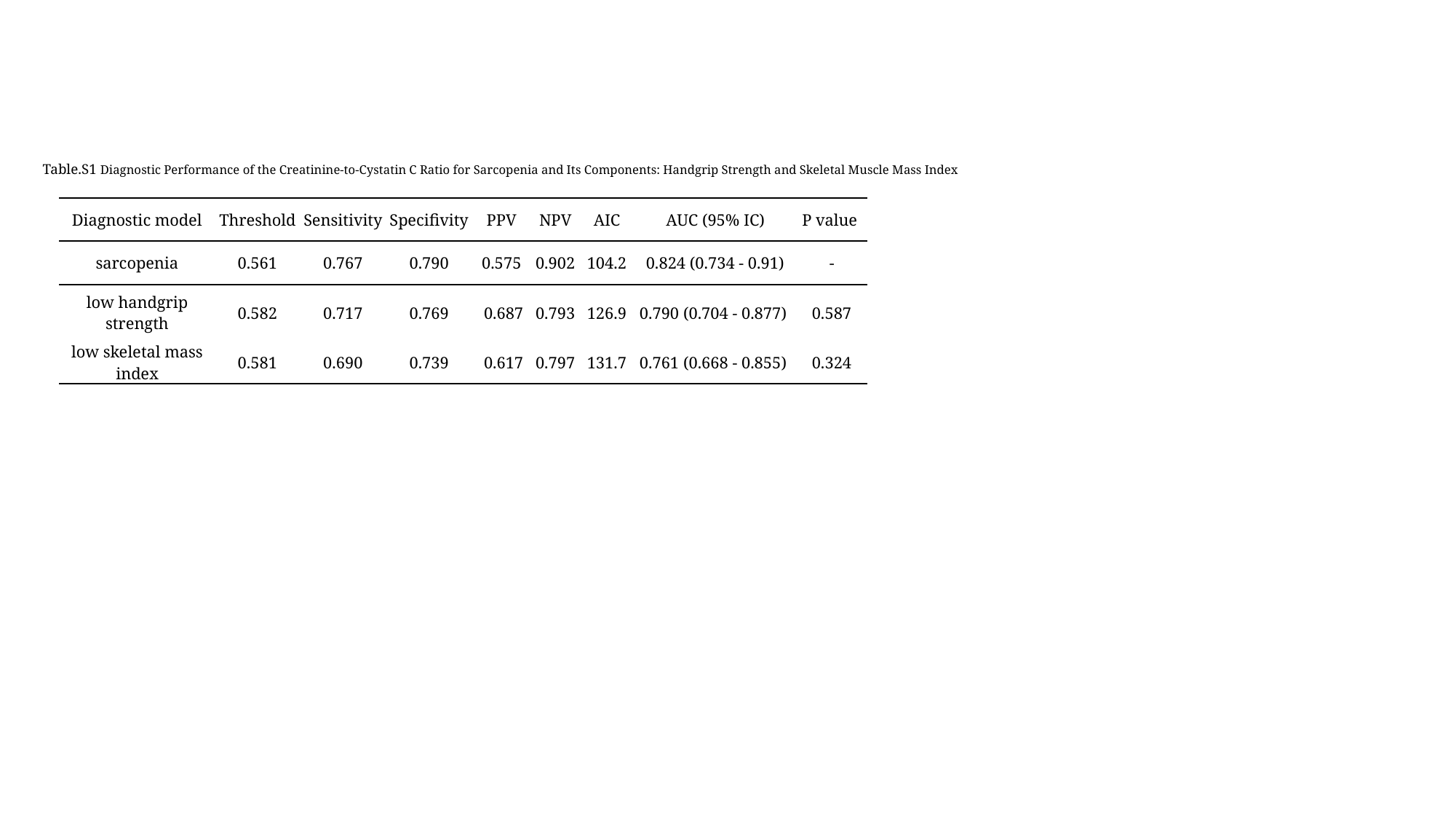

Table.S1 Diagnostic Performance of the Creatinine-to-Cystatin C Ratio for Sarcopenia and Its Components: Handgrip Strength and Skeletal Muscle Mass Index
| Diagnostic model | Threshold | Sensitivity | Specifivity | PPV | NPV | AIC | AUC (95% IC) | P value |
| --- | --- | --- | --- | --- | --- | --- | --- | --- |
| sarcopenia | 0.561 | 0.767 | 0.790 | 0.575 | 0.902 | 104.2 | 0.824 (0.734 - 0.91) | - |
| low handgrip strength | 0.582 | 0.717 | 0.769 | 0.687 | 0.793 | 126.9 | 0.790 (0.704 - 0.877) | 0.587 |
| low skeletal mass index | 0.581 | 0.690 | 0.739 | 0.617 | 0.797 | 131.7 | 0.761 (0.668 - 0.855) | 0.324 |
